# Fear of disease progression, self-management efficacy, and family functioning in patients with breast cancer: a cross-sectional relationship study

**DOI:** 10.3389/fpsyg.2024.1400695

**Published:** 2024-07-09

**Authors:** Jiaru Zhuang, Yuan Wang, Shan Wang, Renjing Hu, Yibo Wu, Ling Chen

**Affiliations:** ^1^Department of Breast Surgery, Affiliated Hospital of Jiangnan University, Wuxi, China; ^2^Department of Laboratory Medicine, Jiangnan University Medical Center, Wuxi, China; ^3^Human Reproductive Medicine Center, Affiliated Hospital of Jiangnan University, Wuxi, China

**Keywords:** breast cancer, fear of progression, self-management efficacy, family functioning, cancer nursing

## Abstract

**Introduction:**

Fear of disease progression (FoP) has been identified as one of the most prevalent unmet needs among breast cancer patients in recent years. The aim of this study was to examine FoP in patients with breast cancer and explore its associations with demographic and clinical characteristics, self-management efficacy, and family functioning. We also aimed to create a clinically-relevant prediction model based off of these factors (i.e., a “nomogram”) to help identify patients’ probability of experiencing high FoP.

**Methods:**

A cross-sectional survey of breast cancer in patients at the Affiliated Hospital of Jiangnan University was conducted from June 2023 to February 2024. The study included the Demographic and Clinical Characteristics Questionnaire, the Fear of Disease Progression Scale (FoP-Q-SF), the Chinese Self-Management Efficacy Scale for Cancer Patients (C-SUPPH), and the Family Care Index Questionnaire (APGAR). Data analysis included descriptive statistics, independent-samples *t*-test, one-way ANOVA, Pearson correlation analysis, and multiple regression analysis. A nomogram was constructed based on multiple regression results and the model performance was evaluated.

**Results:**

A total of 151 breast cancer patients were enrolled in the study. The mean (standard deviation) FoP score of the patients was 35.87 ± 9.24. The average score of C-SUPPH was 96.97 ± 17.29, and the average score of APGAR was 6.74 ± 2.98. Pearson correlation analysis showed that FoP was negatively correlated with self-management efficacy (*r* = −0.544, *p* < 0.01) and family functioning (*r* = −0.730, *p* < 0.01). Multiple regression analysis showed that age (*B* = −4.038), self-management efficacy (*B* = −0.085) and family functioning (*B* = −1.972) were significantly related to FoP, and together explained 36% of FoP variation (*R*^2^ = 0.360, *F* = 20.50, *p* < 0.001). The nomogram of these variables showed satisfactory prediction performance [the Bootstrap Correction Consistency Index (C-index) = 0.872]. According to previous studies, a C-index of >0.70 indicates that the model is acceptable.

**Conclusion:**

We found that greater fear of cancer progression (FoP) was associated with younger age, lower self-management efficacy and poorer family functioning in breast cancer patients. Based on these variables, our exploratory prediction model should be further investigated in order to help identify breast cancer patients who may be at highest risk of experiencing high FoP.

## 1 Introduction

According to the 2023 Global Cancer Data Statistics, breast cancer is the most common malignant tumor among women worldwide (Siegel et al., 2023). In recent years, with significant improvements in adjuvant breast cancer therapy, patients’ life expectancy has increased (Ganz et al., 2024). This means that breast cancer patients are exposed to cancer-related stressors (e.g., fear of progression) for longer periods of time (Sari, 2020). Studies have found that 73% of breast cancer patients experience varying degrees of fear of disease progression, which leads to low compliance with disease treatment, inhibits individual health behaviors, and seriously affects the quality of life (Mehnert et al., 2013). Fear of disease progression, also known as “fear of progress” (FoP) (Lebel et al., 2016) in cancer, is a psychological state in which patients are afraid of the possibility of metastasis or recurrence of the disease. FoP is considered to be one of the most common unmet needs among cancer patients (Ma et al., 2024), highlighting the importance for understanding and addressing this concern.

Numerous earlier studies have examined the factors that influence fear of progression (FoP). Research from several academics has shown that FoP in cancer patients is related to women (Wang et al., 2023), Younger age (Tong et al., 2024), Single (non-married) (Li et al., 2024), and lower income (Richter et al., 2022). High levels of FoP have been found to be correlated with younger age, time since diagnosis, have comorbidities, lower family income, and self-reported counseling in patients with breast cancer in a cross-sectional investigation; however, they were not correlated with sex or cancer stage (Tao et al., 2024). Other studies have shown that breast cancer patients with high psychological scores of fear of disease progression may have worse quality of life and self-management efficacy (Lyu et al., 2023). It is evident that multiple factors impact FoP, which can significantly affect patients’ psychological well-being.

Previous research has shown that patients with high levels of fear of progression (FoP) tend to low levels of self-management efficacy (Dunne et al., 2019). Self-management efficacy is defined as the ability and confidence to perform self-management activities on oneself (Wang et al., 2017). A recent randomized controlled trial showed that implementing a family-centered self-management intervention could significantly reduce FoP levels (Wang et al., 2024). Further research has highlighted the importance of good family functioning in alleviating fear of disease progression and psychological distress in cancer patients (Sawma and Choueiri, 2022). Family function is derived from the special social support of family members and reflects the overall quality of a family’s operation. Indeed, the Mind Sponge Theory suggest that good family functioning is essential in managing fear in cancer patients, providing appropriate emotional support from the family, improving physical health, and reducing the burden of symptoms, which may improve the efficacy of patients’ self-management and thus alleviate fear (Sari, 2019). It can be seen that self-management efficacy and family function are closely related to the FoP levels of breast cancer patients, but the correlation among the three has not been reported. In addition, high levels of FoP not only impair individuals’ social skills, work performance, and quality of life, but also increase the medical economic burden on cancer survivors and the state (Hall et al., 2016; Champagne et al., 2018; Vachon et al., 2020; Rha et al., 2022). Therefore, it is necessary to predict and identify FoP as early as possible, and to provide necessary psychosocial interventions.

Therefore, this study revolves around three hypotheses:

*Hypothesis 1*: There are differences in the degree of fear of disease progression among breast cancer patients based on demographic and disease-related characteristics.

*Hypothesis 2*: Fear of disease progression in breast cancer patients is associated with self-management efficacy and family functioning.

*Hypothesis 3*: A predictive model (i.e., nomogram) for fear of progression (FoP) in breast cancer patients can identify high-risk populations (FoP ≥ 34 points) that have not yet developed high FoP.

## 2 Materials and methods

### 2.1 Study setting and participants

In this study, breast cancer patients who were hospitalized for radiotherapy or chemotherapy at the Affiliated Hospital of Jiangnan University from June 2023 to February 2024 were selected. The inclusion criteria for patients were: (1) previously diagnosed by a clinicopathological histological with breast cancer; (2) clear awareness, good Chinese reading and communication skills; (3) age ≥ 18 years old; (4) agreement and voluntary participation in the study. Exclusion criteria: (1) patients with recurrence or metastasis; (2) other complications, mental disorders, or cognitive impairments; (3) patients with other malignancies Finally, the final sample included 151 breast cancer patients. Informed consent has been obtained from patients for this study.

### 2.2 Ethical consideration

This study was approved by the Ethics Committee of the Affiliated Hospital of Jiangnan University (JNMS04202300125) and the informed consent of the patient has been obtained. The study was conducted in accordance with the guidelines of the Declaration of Helsinki.

### 2.3 Data collection

Researchers were supported by their hospitals and departments and selected study subjects strictly according to inclusion and exclusion criteria. Prior to the survey, the purpose, significance, and content of the survey were explained to the study subjects. Trained investigators use uniform questionnaires and conduct face-to-face surveys to gather information from participants. A total of 160 questionnaires were distributed, and 9 patients had to stop the questionnaire due to physical discomfort. In the end, a total of 151 complete questionnaires were collected.

### 2.4 Demographic and disease-related characteristics

A self-designed questionnaire was used to collect sociodemographic characteristics such as age, marital status, education level, *per capita* monthly household income, place of residence (city/countryside), religious belief (yes/no), current treatment (chemotherapy/radiotherapy), surgical method, payment method of medical expenses, and disease stage.

### 2.5 Simplified Chinese scale for fear of disease progression in cancer patients

This scale was used to investigate the level of fear in breast cancer patients in China (Hinz et al., 2014). The scale consists of two dimensions: physiological well-being (6 items) and social/family (6 items), with a total score of 12 items ranging from 1 (“never”) to 5 (“always”). Therefore, the total score ranges from 12 to 60 points, with higher scores indicating more severe levels of fear of progression (FoP). A total score of ≥34 indicates that the subject has a high level of FoP (Hinz et al., 2014). The Chinese version of the scale had a Cronbach’s α coefficient of 0.883, demonstrating good reliability and validity.

### 2.6 Chinese version of cancer patients’ self-management efficacy scale

The Strategies Used by People to Promote Health (SUPPH), which was developed by Lev and Owen (1996) and Qian and Yuan (2011) named it as “Chinese version of the self-management efficacy scale for cancer patients” (C-SUPPH) in 2011 to C-SUPPH includes three dimensions: self-stress reduction (three items), positive attitude (15 items), and self-decision (10 items), with a total of 28 items. The scale adopts a 1-5point scoring method, where 1 point represents “no confidence” and 4 points represent “very confident.” The total score ranges from 28 to 140 points, with a higher score indicating higher patient self-management efficacy. According to the score range, scores≤65 indicate low self-management efficacy, scores between 66 and 102 indicate moderate level of sufficient self-management efficacy, and scores ≥ 103 indicate high self-management efficacy. The C-SUPPH has good reliability and validity, with a Cronbach’s alpha coefficient of 0.970 and a test–retest reliability of 0.937.

### 2.7 Family care index questionnaire

The Family Care Index was designed and developed by Smilkstein (1978) of the University of Washington in Seattle, United States. It is used to evaluate five aspects: family fitness, cooperation, maturity, affectivity, and intimacy, hence it is referred to as the Family APGAR Index. Each item of APGAR is scored on a 3-level scale (never = 0 points, sometimes = 1 point, often = 2 points), with a total score of 0–10 points. The higher the score, the better the family functioning. According to the scoring range, 0–3 points indicate severe impairment of family functioning, 4–6 points indicate moderate impairment, and 7–10 points indicate good family functioning. The APGAR has good reliability and validity, with a Cronbach’s α coefficient of 0.780 and a test–retest validity of 0.800.

### 2.8 Data analysis

The nomogram were performed by the R programming language and environment version 4.0.2.^1^ Other statistical analyses were conducted using IBM Statistical Package for Social Sciences (SPSS) version 26.0. All statistical tests were two-sided and *p* < 0.05 was regarded as statistically significant. Descriptive statistics are expressed in frequency and percentage. Variance analysis selects parametric class tests. Independent samples *t*-test and one-way ANOVA were used to compare differences between groups. Pearson correlation analysis was used to explore the relationship between fear of disease progression, self-efficacy, and family care in breast cancer patients. Multiple regression analysis was used to determine the factors influencing the fear of disease progression in breast cancer patients, and a nomogram was constructed to predict the fear of disease progression based on the results.

A nomogram is developed based on the results of multivariate regression analysis, merging multiple influencing factors. This involves plotting line segments with scales on the same plane at a certain scale to demonstrate the relationship between variables in the prediction model (Huang et al., 2023). The nomogram is generated using the R programming language. The bootstrap resampling method was used to verify the prediction accuracy of nomogram, with Correction Consistency Index (C-index) > 0.70 indicating that the model is acceptable (Harrell et al., 1984).

## 3 Results

### 3.1 Descriptive analysis

The 151 patients were aged 26–79 years (SD = 12.53), with an average age of 54.38 years. 63.6% (*n* = 96) of the patients were younger than 60 years, and 36.4% (*n* = 55) were older than 60 years. 97.4% (*n* = 147) of the patients were married, and 23.8% (*n* = 36) had a university degree or higher. Table 1 shows illustrative statistics and correlations among specific demographic data, clinical characteristics, and fear of progression (FoP).

**Table 1:** Univariate analysis of FoP in breast cancer patients (*N* = 151).

Characteristics	Cases (*n*)	Percentage (%)	Fear disease progression score	*F/t*	*p*
Age (years)				2.10	0.038
<60	96	63.6	36.97 ± 9.95		
≥60	55	36.4	33.95 ± 7.57		
Medical insurance				1.941	0.054
Yes	144	95.4	35.55 ± 9.16		
No	7	4.6	42.43 ± 9.22		
Educate				1.124	0.341
Primary school and below	40	26.5	35.63 ± 6.26		
Middle school	43	28.5	36.37 ± 10.35		
High school	32	21.2	37.81 ± 10.98		
College degree or above	36	23.8	33.81 ± 8.89		
Marriage				1.071	0.286
Yes	147	97.4	35.73 ± 9.31		
No	4	2.6	40.75 ± 4.50		
Monthly household income				6.833	0.001
<1,000	28	18.5	38.86 ± 6.71		
1,000–2,999	82	54.3	39.96 ± 10.00		
≥3,000	41	27.2	31.63 ± 7.77		
Residence				−1.874	0.063
City	83	55.0	34.60 ± 9.31		
Countryside	68	45.0	37.41 ± 8.99		
Religious belief				−0.743	0.458
Yes	8	5.3	33.50 ± 10.23		
No	143	94.7	36.00 ± 9.21		
Cancer stage				7.950	0.001
I	49	32.5	32.02 ± 8.63		
II	78	51.7	37.00 ± 8.55		
III	24	15.9	40.04 ± 10.16		
Surgical method				0.918	0.401
Radical mastectomy	66	43.7	36.23 ± 9.02		
Breast conserving surgery	44	29.1	34.34 ± 10.49		
Simple resection	41	27.2	36.93 ± 8.13		
Current treatment				−1.247	0.214
Chemotherapy	105	69.5	35.25 ± 9.40		
Surgery	46	30.5	37.28 ± 8.82		

### 3.2 Univariate analysis

There were significant differences in age, income, and cancer stage among FoP (*p* < 0.05), but there were no significant differences in FoP in terms of type of medical insurance, education level, marital status, place of residence, religious belief, surgical method, and current treatment (*p* > 0.05).

### 3.3 Correlation analysis

As shown in Table 2, the average score for FoP is 35.87 ± 9.24, indicating that FoP is at a moderately high level. The average rating for C-SUPPH is 96.97 ± 17.29 and the average rating for APGAR is 6.74 ± 2.98. In addition, Table 3 shows that FoP is negatively correlated with self-management efficacy (*r* = −0.544, *p* < 0.01) and negatively correlated with family functioning (*r* = −0.730, *p* < 0.01). Self-management efficacy is positively correlated with family functioning (*r* = 0.607, *p* < 0.01). This further suggests that the higher the level of self-management efficacy, the lower the FoP level, and the higher the level of family functioning, the lower the patient’s FoP level.

**Table 2:** Specific descriptive analysis of FoP, APGAR, and C-SUPPH scores (*N* = 151).

Parameter	Score range	Average ± SD
FoP scale score (total)	13–53	35.87 ± 9.24
Physiological health	6–28	20.08 ± 4.84
Social family	6–26	15.79 ± 5.04
C-SUPPH scale score (total)	60–129	96.97 ± 17.29
Positive attitude	29–68	51.32 ± 9.86
Self-decompression	15–47	35.65 ± 6.68
Self-decision-making	4–15	10.00 ± 2.47
APGAR scale score (total)	2–10	6.74 ± 2.98
Adaptability	0–2	1.42 ± 0.60
Partnerships	0–2	1.32 ± 0.75
Grow	0–2	1.31 ± 0.76
Emotional	0–2	1.36 ± 0.70
Intimacy	0–2	1.33 ± 0.70

FoP, Fear of Progress; C-SUPPH, Self-management Efficacy Scale for Cancer Patients; APGAR, Family Care Index.

**Table 3:** Correlation of subjects FoP, APGAR and C-SUPPH (*n* = 151).

	Patient’s FoP	APGAR	C-SUPPH
Patient’s FoP	1	−0.730**	−0.544**
APGAR	−0.730**	1	0.607**
C-SUPPH	−0.544**	0.607**	1

**p < 0.01; FoP, Fear of Progress; C-SUPPH, Self-management Efficacy Scale for Cancer Patients; APGAR, Family Care Index.

### 3.4 Regression analysis

According to the results of the t-test, ANOVA, and Pearson correlation analysis, significant variables were input into the regression equation model. As shown in Table 4, age (*B* = −4.038), self-management efficacy (*B* = −0.085), and family functioning (*B* = −1.972) were significantly associated with of fear of progression (FoP) in breast cancer patients. These variables represented 36% of the variance, and the analysis results were statistically significant (*R*^2^ = 0.360, *F* = 20.50, *p* < 0.001).

**Table 4:** Multiple regression results with FoP as dependent variables (*N* = 151).

Variable	*B*	Sx	*T*	*p*
Constant	43.385	4.301	10.087	<0.001
Age	−4.038	1.480	−2.728	0.007
Stage	0.009	0.050	0.188	0.851
Monthly income	−0.031	0.052	−0.594	0.554
C-SUPPH	−0.085	0.037	−2.288	0.024
APGAR	−1.972	0.217	−9.101	<0.001

*R*^2^ = 0.360; *R*_ad_ = 0.342; *F* = 20.50; *p* < 0.001. FoP, Fear of Progress; C-SUPPH, Self-management Efficacy Scale for Cancer Patients; APGAR, Family Care Index.

### 3.5 A nomogram predicting the probability of progression of the fear disease

**Figure 1:**
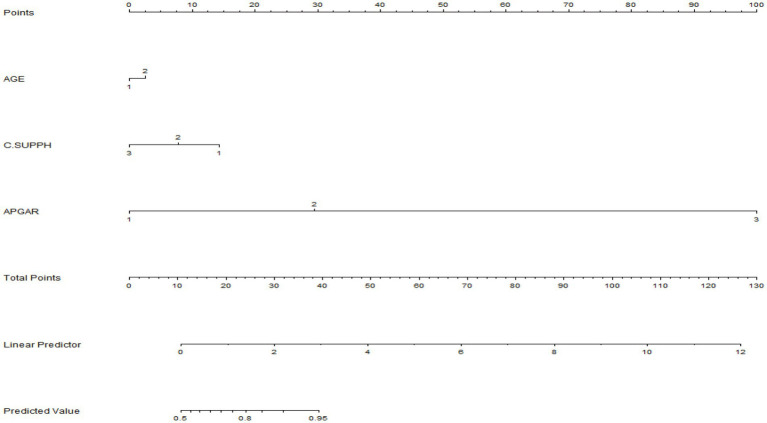
A nomogram predicting the probability of progression of the fear disease.

Based on the results of multiple regression, we constructed a nomogram to predict the patient’s risk of experiencing FoP is based on risk factors. In Figure 1, the left side shows the variable names. For example, “age (1/2)” represents “age ≥ 60 years” or “age < 60 years,” respectively. The C-SUPPH score (1/2/3) is expressed as “low/medium/high self-management efficacy,” respectively. The APGAR score (1/2/3) represents “family functioning (poor/moderate/good),” respectively. For instance, a breast cancer patient who is 50 years old, has a C-SUPPH score of 80 points (moderate), and an APGAR score of 6 points (moderate). In the nomogram, each variable is assigned a specific score on the rating scale. Therefore, “age (50 years)” = 2.5 points, “C-SUPPH (intermediate level)” = 7.5 points, “APGAR (intermediate level)” = 27.5 points, and the total score is 2.5 + 7.5 + 27.5 = 37.5 points. Therefore, a total score of 37.5 corresponds to a prediction probability of about 90%. In this case, the breast cancer patient has an 90% risk of developing high FoP.

The prediction accuracy of the nomogram was internally verified by the bootstrap resampling method (1,000 bootstrap resamples), and the Bootstrap Correction Consistency Index (C-index) of the nomogram was 0.872. Our prediction model has good predictive value, which can make a relatively accurate assessment of patients, which helps to identify risk factors early and reduce FoP.

## 4 Discussion

### 4.1 Current status of FoP among breast cancer patients

The results of this study showed 62.9% of the patients experienced moderately high levels of fear of progression (FoP), which was similar to the results of Wang et al. (2023). However, there was a higher incidence of FoP in breast cancer patients compared to previous reports in prostate cancer and non-small cell lung cancer. This may be related to the type of cancer and treatment modality (Bergerot et al., 2021; Liu et al., 2022). Given the frequency of FoP we observed in our sample, resources and supportive psychosocial services that could help reduce FoP and promote adaptive coping strategies should be made available to breast patients (especially younger patients) by their medical team.

### 4.2 Demographic associations with FoP for breast cancer patients

Our findings suggest that age is significantly correlated with levels of fear of progression (FoP), with younger age being associated with higher FoP levels. This is consistent with previous studies that show that women who are diagnosed with breast cancer at a younger age experience more psychological distress as compared to women diagnosed later in life (Vandraas et al., 2021). In general, young women are subject to more family and social responsibilities, as well as pressure to support their elders and children (Tao et al., 2024). Breast cancer patients may also face difficulty finding employment and financial hardship after developing cancer (Naughton et al., 2020). Some patients may be unable to continue meeting their obligations to support their parents and children (Meeker et al., 2017).

### 4.3 The relationship between self-management efficacy and FoP

This study found that fear of progression (FoP) in breast cancer patients can significantly associated with self-management efficacy. This is similar to the result reported by Jayakumar et al. (2024) of low self-management efficacy in patients with high levels of fear of pain. When faced with negative emotions that arise during the treatment of the disease, patients with high self-management efficacy believe that they will be able to overcome the disease and externalize this belief into action by actively seeking medical treatment. Dawson et al. (2016) showed that patients with high self-management efficacy were more likely to express their worries and fears to healthcare workers, which was effective in relieving psychological stress. Even if cancer recurs, confiding could help patients cope calmly, thereby reducing the fear of recurrence. It is recommended that medical staff carry out psychological counseling interventions for inpatients with breast cancer to stimulate patients’ potential beliefs, help them improve their self-management efficacy, and reduce their fear of recurrence.

### 4.4 The relationship between family functioning and FoP

Our results showed that family functioning was an influencing factor for fear of disease progression in breast cancer patients, and it was negatively correlated with fear of disease progression. Family functioning, or a positive social environment from a family source, may intervene with the information provided around the family. Tightening family restraints in life crisis situations, as what happens to family caregivers and patients in cancer care can have a positive impact on information filtering activities, as positive information circles produce positive psychological products (low FoP). Family functioning can effectively reflect family members’ satisfaction with intimacy, communication, and emotional support (Roberts and Ishler, 2018; Beckmeyer and Russell, 2022). Studies have shown that good family functioning is a protective factor for couples’ mental health, as it can prevent family members from developing negative emotions (Sidek et al., 2022). Therefore, medical staff can encourage spouses to actively communicate with family members and express difficulties and ideas. This will allow them to fulfill their family functions, reduce the burden on spouses, and better help them adapt to changes in family structure.

### 4.5 Construction and evaluation of psychological prediction model for fear of disease progression in breast cancer patients

Currently, there is no effective clinical prediction model for breast cancer FoP, and the model cannot accurately predict individuals. In this study, a nomogram was constructed based on three variables. All variables were measured using scales of high reliability and validity, except for age. The Bootstrap resampling method was used to verify the prediction accuracy of the nomogram, which confirmed the accuracy of the model in predicting FoP (C-index = 0.872). According to previous studies, a C-index of >0.70 indicates that the model is acceptable (Harrell et al., 1984). Unfortunately, our model has not yet been validated in an external population, and the external validity of the model may require further confirmation.

## 5 Strengths and study limitations

The advantages of this research are as follows: First, by investigating the level of disease fear in breast cancer patients, and exploring its influencing factors, it will provide a basis for research related to disease progression fear. Second, so far, there has been no prediction model that specifically explores FoP using multiple regression methods for fear disease progression, and our study can provide a reference for it.

Nevertheless, this research has certain limitations that need to be acknowledged: First, FoP levels in breast cancer patients fluctuate over time, and the influencing factors may vary, so longitudinal studies are needed to explore the FoP trajectory of breast cancer patients. Second, participants’ FoP, self-management efficacy, and family functioning were self-reported survey data, which may be susceptible to recall biases. Finally, the sample size is relatively small, which may affect the generalizability of the findings, and other variables that may affect FoP have not been studied. Therefore, we will conduct multicenter studies and expand the sample size in the future.

## 6 Conclusion

The survey found that FoP is common in breast cancer patients. In this study, self-management efficacy and family functioning were inversely correlated with FoP in breast cancer patients. The nomogram constructed based on the existing population helps health care providers quickly and accurately identify the high fear of cancer recurrence in high-risk individuals, so as to develop timely, predictable, and personalized intervention plans for high-risk patients in high-risk populations.

## Data availability statement

The raw data supporting the conclusions of this article will be made available by the authors, without undue reservation.

## Ethics statement

The studies involving humans were approved by the Ethics Committee of the Affiliated Hospital of Jiangnan University (JNMS04202300125). The studies were conducted in accordance with the local legislation and institutional requirements. The participants provided their written informed consent to participate in this study.

## Author contributions

JZ: Writing – original draft, Software, Data curation, Conceptualization. YuW: Writing – original draft, Project administration, Methodology. SW: Writing – original draft, Validation, Software, Investigation. RH: Writing – original draft, Visualization, Validation, Data curation. YiW: Writing – review & editing, Supervision, Funding acquisition, Conceptualization. LC: Writing – review & editing, Supervision, Project administration, Funding acquisition.

## Footnotes

^1^http://cran.r-project.org
