# Supplementary material for: Fear of disease progression, self-management efficacy, and family functioning in patients with breast cancer: a cross-sectional relationship study
Source: Front Psychol. 2024 Jul 9;15:1400695. doi: 10.3389/fpsyg.2024.1400695 (PMC11264380; doi:10.3389/fpsyg.2024.1400695)
Supplement: Supplementary file 1 [file Data_Sheet_1.docx]

**Chinese version of the Cancer Self-Management Efficacy Scale**

Instructions: This scale is used to evaluate your confidence in dealing with illness and to help your health care provider understand how you are dealing with your illness. There is no right or wrong answer, please type "√" in the corresponding grid according to your actual situation.

| 1=No faith 2=A little bit of faith 3=Confidence 4=Very confident 5=Very confident | | | | | |
| --- | --- | --- | --- | --- | --- |
| entries | 1 | 2 | 3 | 4 | 5 |
| 1. Exclude distressing thoughts from my consciousness |  |  |  |  |  |
| 2. Use relaxation to reduce my anxiety |  |  |  |  |  |
| 3. Be proactive in finding ways to relieve stress |  |  |  |  |  |
| 4. Do things to help overcome current emotional distress |  |  |  |  |  |
| 5. Even when I'm feeling bad, I try to relieve stress |  |  |  |  |  |
| 6. Try to manage the anxiety associated with the illness and not let it progress to uncontrollable levels |  |  |  |  |  |
| 7. Think about the fact that people younger than me are sick and in good health |  |  |  |  |  |
| 8. Reduce my anxiety by focusing on things that are not related to my illness |  |  |  |  |  |
| 9. Believe that stress can be controlled |  |  |  |  |  |
| 10. Be able to choose a suitable treatment method from the doctor's recommendation |  |  |  |  |  |
| 11. Be able to make choices about your own treatment plan |  |  |  |  |  |
| 12. Be able to decide whether to receive or not to receive treatment |  |  |  |  |  |
| 13 Since he was sick, he still felt the joy of life |  |  |  |  |  |
| 14. Being able to do something special for yourself to make life better |  |  |  |  |  |
| 15. Believe that you can cope with the stress of illness |  |  |  |  |  |
| 16. Do what you can to help other patients through illness and treatment |  |  |  |  |  |
| 17. Believe you're not that bad yet |  |  |  |  |  |
| 18. Keep your stress under control |  |  |  |  |  |
| 19. Focus on other important things in life |  |  |  |  |  |
| 20. Believe that I can find my inner strength, faith, will |  |  |  |  |  |
| 21. Believe that you will overcome the disease |  |  |  |  |  |
| 22. Find a way to help me through this difficult time |  |  |  |  |  |
| 23. I have a positive attitude towards my state of health |  |  |  |  |  |
| 24. Doing things helps me overcome my current physical problems |  |  |  |  |  |
| 25 Do things to control my weariness |  |  |  |  |  |
| 26. If I feel depressed, I find a way to adjust myself |  |  |  |  |  |
| 27. Be able to actively cope with the side effects of treatment |  |  |  |  |  |
| 28. Be able to cope with the frustration of illness and treatment |  |  |  |  |  |
