# Supplementary material for: Fear of disease progression, self-management efficacy, and family functioning in patients with breast cancer: a cross-sectional relationship study
Source: Front Psychol. 2024 Jul 9;15:1400695. doi: 10.3389/fpsyg.2024.1400695 (PMC11264380; doi:10.3389/fpsyg.2024.1400695)
Supplement: Supplementary file 2 [file Data_Sheet_2.docx]

**The Chinese version of the Simplified Scale for the Progression of Fear Disease**

Guidance: The following may be your worries or concerns, please type "√" in the corresponding grid according to your feelings, and there is no right or wrong answer.

| entries | never | seldom | sometimes | often | always |
| --- | --- | --- | --- | --- | --- |
| 1. Fear that the disease will progress |  |  |  |  |  |
| 2. I feel nervous before the doctor's check-up and regular check-ups |  |  |  |  |  |
| 3. Fear of pain |  |  |  |  |  |
| 4. Worry about the possibility of affecting future work |  |  |  |  |  |
| 5. Have some symptoms (such as rapid heartbeat, stomach pain, etc.) |  |  |  |  |  |
| 6. Fear that my child will catch the disease |  |  |  |  |  |
| 7. Worry about relying on strangers for future activities and daily life |  |  |  |  |  |
| 8. I'm worried that I won't be able to pursue my hobbies in the future |  |  |  |  |  |
| 9. Worry that there will be some big treatment in the course of the disease |  |  |  |  |  |
| 10. Fear that treatments and medications will damage my body |  |  |  |  |  |
| 11. Worry about what will happen to the family if something happens to me |  |  |  |  |  |
| 12. I am worried that I will not be able to continue working in the future |  |  |  |  |  |
