# Supplementary material for: Fear of disease progression, self-management efficacy, and family functioning in patients with breast cancer: a cross-sectional relationship study
Source: Front Psychol. 2024 Jul 9;15:1400695. doi: 10.3389/fpsyg.2024.1400695 (PMC11264380; doi:10.3389/fpsyg.2024.1400695)
Supplement: Supplementary file 3 [file Data_Sheet_3.docx]

**Family Care Index Questionnaire**

Instructions: Please put "√" on the appropriate form for your relationship with your family

| entries | often | Sometimes | Almost rarely |
| --- | --- | --- | --- |
| 1. When I have a problem, I can get satisfactory help from my family |  |  |  |
| 2. I am satisfied with the way my family discusses various things with me and shares the problem |  |  |  |
| 3. When I engage in new activities or developments, my family is receptive and supportive |  |  |  |
| 4. I am satisfied with the way my family shows concern and love for my emotions |  |  |  |
| 5. I'm happy with the way my family spends time with me |  |  |  |
